# The Effect of Slow Electrical Stimuli to Achieve Learning in Cultured Networks of Rat Cortical Neurons

**DOI:** 10.1371/journal.pone.0008871

**Published:** 2010-01-25

**Authors:** Joost le Feber, Jan Stegenga, Wim L. C. Rutten

**Affiliations:** MIRA: Institute for Biomedical Engineering, Department of Electrical Engineering, Mathematics, and Computer Science, University of Twente, Enschede, The Netherlands; Center for Genomic Regulation, Spain

## Abstract

Learning, or more generally, plasticity may be studied using cultured networks of rat cortical neurons on multi electrode arrays. Several protocols have been proposed to affect connectivity in such networks. One of these protocols, proposed by Shahaf and Marom, aimed to train the input-output relationship of a selected connection in a network using slow electrical stimuli. Although the results were quite promising, the experiments appeared difficult to repeat and the training protocol did not serve as a basis for wider investigation yet. Here, we repeated their protocol, and compared our ‘learning curves’ to the original results. Although in some experiments the protocol did not seem to work, we found that on average, the protocol showed a significantly improved stimulus response indeed. Furthermore, the protocol always induced functional connectivity changes that were much larger than changes that occurred after a comparable period of random or no stimulation. Finally, our data shows that stimulation at a fixed electrode induces functional connectivity changes of similar magnitude as stimulation through randomly varied sites; both larger than spontaneous connectivity fluctuations. We concluded that slow electrical stimulation always induced functional connectivity changes, although uncontrolled. The magnitude of change increased when we applied the adaptive (closed-loop) training protocol. We hypothesize that networks develop an equilibrium between connectivity and activity. Induced connectivity changes depend on the combination of applied stimulus and initial connectivity. Plain stimuli may drive networks to the nearest equilibrium that accommodates this input, whereas adaptive stimulation may direct the space for exploration and force networks to a new balance, at a larger distance from the initial state.

## Introduction

**Figure 1:**
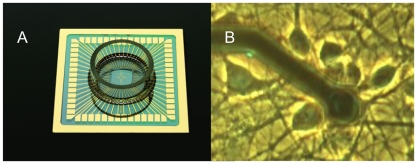
Multi electrode array (MEA) and close up of one of the electrodes. **A**: MEA, used to record neuronal activity in cultured networks of cortical neurons. It is based on a glass substrate with 60 embedded electrodes in the centre of the chamber, with 100 µm inter electrode distance. The glass ring glued on top was filled with glia conditioned growth medium and firmly sealed. **B**: close up of one of the electrodes and several neurons. Electrode diameter: 10 µm. Most electrodes did not pick up signals from more than one neuron.

Whereas the formation and development of connections is assumed to be crucial in the process of learning, their conservation is possibly essential for memory. Assuming that network connections are reflected in the patterns of electrical activity, connectivity studies often entail simultaneous measurement of activity in a large number of neurons. To facilitate access to such a large number of neurons, several groups now use preparations of cultured neurons grown over a multi electrode array (MEA, see Figure 1). This enables simultaneous measurement from multiple electrodes, as well as network manipulation using selective electrical stimulation.

Several studies investigated the development of neuronal connections using various methods to induce plasticity [1]–[7]. All of these methods were based on the hypothesis that certain patterns of activity may change synaptic efficacy. Although some results appeared quite successful, other experiments yielded ambiguous results or were difficult to reproduce [5], [8] An important complicating factor is the high variability in spontaneous activity patterns in cultured cortical networks, which may mask induced alterations. Spontaneous activity shows alternating periods of seemingly uncorrelated firing at some electrodes and of short synchronized firing at many electrodes, usually referred to as network bursts [9], [10] These network bursts often comprise many action potentials within a time window that has been shown to induce spike timing dependent plasticity [11]–[14], and may therefore influence network connectivity. Thus, induced connectivity changes may go undetected among the large spontaneous fluctuations, or may disappear again, due to the fixed strong embedded patterns of bursting, hampering detection of changes in a selected connection. Therefore, the probability to observe induced connectivity changes may be largely increased using a larger network-wide scale of monitoring.

**Figure 2:**
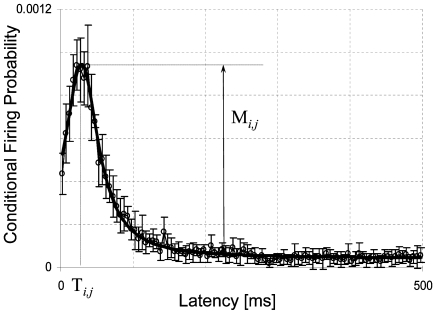
Example of estimated conditional firing probability (○, mean±SD of 5 consecutive bins of 0.5ms each). Solid line represents fitted equation, used to obtain values for strength (M*_i,j_*) and latency (T*_i,j_*) of the functional connection between a pair of electrodes (*i,j*).

To study connectivity in a larger part of the network, we used conditional firing probability (CFP) analysis [15]. CFP analysis reveals relationships between pairs of electrodes, characterized by two parameters: strength and latency. Figure 2 shows an example of a CFP curve and the calculated strength and latency. CFP analysis is related to cross-correlation, and provides descriptions of functional connections, abstract representations of neuronal pathways between neuron pairs [16]. Functional connectivity is model free, that is, it measures statistical interdependence without explicit reference to causal effects [17]. A recent study suggests that functional connections, at least to a certain extend, describe anatomical connectivity (the set of physical or structural (synaptic) connections linking neuronal units at a given time) because they follow the rules of spike timing dependent plasticity [18]. Together, all CFPs yield a *functional connectivity matrix*, containing strengths and latencies of all functional connections in the network in a certain time interval. Temporal sequences of connectivity matrices may then be used to investigate the development of network connectivity.

Most plasticity studies did not aim to alter connectivity in a predefined way, or with a specific goal. However, one protocol, by Marom and Shahaf [3] used activity dependent adaptive stimulation, aiming to train a culture to produce a predefined response upon stimulation. They based their approach on general learning theories they referred to as ‘stimulus regulation principle’. In their experiments, the reward acted to reduce the driving stimulus, precluding the acquisition of any new stimulus-response associations. Thus, no separate neural rewarding entity is postulated or needed for shaping behavior [19]. Although their results seemed quite successful, they appeared difficult to reproduce and they did not serve as a basis for wider exploration yet. One study that did succeed to reproduce the results of [3] in cultures of hippocampal neurons, reported that the protocol was successful only in part of their experiments [20] These results were in agreement with our own observations [21] This latter study showed that the success rate drops even further if more than the first ∼10 trials that were evaluated in [3] and [20] are taken into account. It also showed that not only burst profiles changed significantly, but also phase profiles, indicating that the contributions from individual electrodes (neurons) changed significantly. Although this change was larger in electrodes closely related to the ones selected for stimulation and evaluation, plasticity occurred on network level, showing that a change in a simple input-output relationship between two neurons required network wide connectivity changes.

It is not completely understood how and why slow electrical stimulation (f_stim _<1 Hz) may alter network connectivity. A recent study suggested that low frequency stimuli produced neither short- nor long-term changes in the evoked response of networks [7]. Another study showed that repeated slow stimulation at single electrodes (40 pulses per electrode, delivered through 6 electrodes) transformed an initially stable pattern of stereotypical spontaneous activity into another activity pattern that remained stable for at least one hour in all cultures. However, the cultures differed with respect to how their activity was modified. Thus, slow stimuli may indeed change connectivity, but not in a controlled manner [6]. Moreover, their study also suggested that such stimuli affect activity in the whole network.

In this study we investigated the influence of slow electrical stimuli on network functional connectivity in more detail. Is it possible indeed to influence network connectivity using single pulse stimulation at a low frequency? Does it make a difference whether we use a single permanent electrode for stimulation, or vary randomly over all electrodes? What is the added value of adaptive stimulation as in the training protocol? To answer these questions, we used three different protocols: random stimulation, single electrode stimulation, and adaptive single electrode stimulation as proposed in [3] The effects of each stimulus protocol were assessed by changes in the functional connectivity matrix, which were compared to each other and to spontaneously occurring changes during periods of equal duration. Our results show that low frequency electrical stimulation may indeed affect functional connectivity, and that adaptive stimulation yields changes of significantly larger magnitude than activity independent stimulation.

## Methods

### A. Cell Cultures

We obtained cortical cells from newborn Wistar rats at post natal day 1. After trypsin treatment cells were dissociated by trituration. About 400,000 dissociated neurons (400 µl suspension) were plated on a 60 electrode MEA (Multi Channel Systems, Reutlingen, Germany, see Figure 1), precoated with poly ethylene imine (PEI). This procedure resulted in an initial cell density of approximately 5000 cells per mm^2^, which was in agreement with counted estimates in the first days after plating. We used MEA's containing electrodes with 10 µm diameter (pitch 100 µm), or 30 µm diameter (pitch:200 µm)

Neurons were cultured in a circular chamber with inner diameter d = 20mm, glued on top of an MEA. The culture chamber was filled with ∼700 µl R12 medium [22] MEAs were stored in an incubator, under standard conditions of 37°C, 100% humidity, and 5% CO_2_ in air. For recording, we firmly sealed the culture chambers with watertight but CO_2_ permeable foil (MCS; ALA scientific), and placed the cultures in a measurement setup outside the incubator. During recording we maintained the CO_2_ level of the environment around 5% and we moisturized the air. For details about the recording setup see [23] All recordings were started after an accommodation period of at least 20 minutes.

After the measurements the cultures were returned to the incubator. We used 16 neuronal cultures obtained from puppies from 16 different rats for 40 experiments (see Table 1), which were performed 32±16 days after plating of the dissociated cells.

**Table 1:** Effects of various protocols on network connectivity.

Experiment:	N	Stimulation period [min]	FSCS	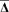	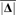	PI	Age [DIV]
Training	10	317±160	0.65±0.24	−0.19±0.49	0.55±0.33	0.43±0.29*^1,2^*	26±14
Random electrode	4	300±0	0.35±0.10	0.16±0.41	0.57±0.29	0.20±0.10*^1^*	41±24
No stimulation	7	300±0	0.34±0.14	−0.05±0.17	0.28±0.10	0.10±0.08*^2^*	34±4
Random electrode	6	67±16	0.30±0.10	0.11±0.21	0.38±0.10	0.12±0.06*^3^*	41±27
Single electrode	6	73±52	0.23±0.09	−0.24±0.16	0.41±0.04	0.10±0.05	27±2
No stimulation	7	61±1	0.16±0.14	−0.16±0.28	0.32±0.21	0.05±0.06*^3^*	34±4

N: No. experiments; FSCS: Fraction of functional connections with Significantly Changed Strength; 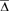: mean strength change of significantly affected functional connections.; 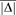: mean *absolute* strength change of significantly affected connections. PI: plasticity index. PI = 1 means that the strength of all functional connections changed by 100%; PI = 0 means no connectivity changes. All values are expressed as mean ± SD. The first three rows show results from ∼5 hour protocols, the last three rows describe ∼1 hour protocols. A ∼1 (or ∼5) hours protocol means that a *stimulation period* lasted for ∼1 (or ∼5) hours. These were preceded and followed by spontaneous recordings and therefore complete experiments always lasted longer than one (or five) hours.

The plasticity index (PI) of the training protocol was significantly larger than those of random or no stimulation (*^1,2^*: t-test, p<0.05). The difference between (5 hour) Random- and no stimulation was not significant (p = 0.08). Using 1 hour protocols the difference between random and no stimulation was significant (*^3^*: p<0.05), the difference between single electrode- and no stimulation was not (p = 0.08). All plasticity indices, except after 1 hour without stimulation, were significantly larger than 0 (p<0.05).

All research involving animals has been conducted according to Dutch law (as stated in “Wet op de dierproeven”), and approved by DEC, the Dutch Animal Use Committee.

### B. Training Experiments

**Figure 3:**
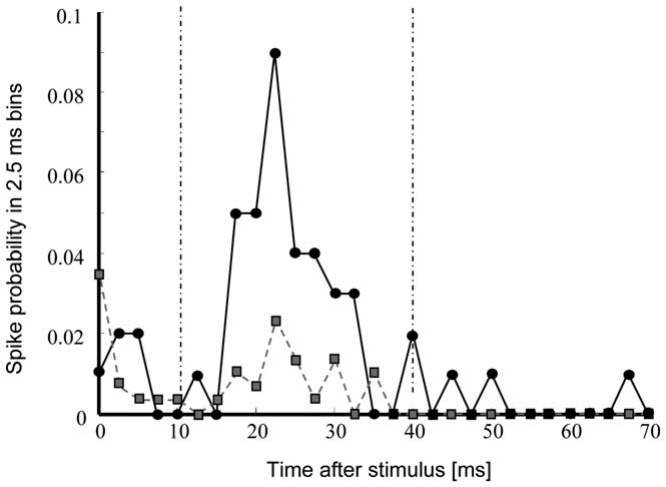
Effect of training protocol on the post stimulus time histogram (responsiveness) of the evaluation electrode. Dashed (gray, ▪) line shows the probability to record an action potential at a selected evaluation electrode during the first 10 stimuli of the training protocol. The time interval to determine the ‘responsiveness’ (the fraction of stimuli that yielded at least one action potential in this interval) was set at 10–40 ms (dash-dotted lines), such that the summed probability before training was about 0.1. Solid (black, •) line: same probability during the last 10 stimuli of the training protocol.

We used biphasic current pulses (200 µs per phase, negative first) at a low frequency (0.2–0.33Hz) to stimulate the cultures. We stimulated all electrodes in random order, at various amplitudes to select a stimulation electrode and amplitude that frequently induced a network burst. Then, following the original training protocol [3], we selected an evaluation electrode that responded to these stimuli at a ratio of ∼0.1. For each electrode we plotted the post stimulus time histogram (PSTH's; curves of the number of action potentials at each electrode, as a function of the latency to the stimulus). Figure 3 shows an example of the probability to record an action potential at the evaluation electrode as a function of the latency after the stimulus (the ‘responsiveness’ of a selected electrode). Response curves of evaluation electrodes usually had a peaked shape, similar to that in Fig. 3. The first peak around zero latency was probably caused by some residual stimulus artifact, or by non-synaptically transmitted direct responses [24] through retrograde stimulation of axons. We focused on the second peak, around 20 ms in the example in Fig. 3.

We selected evaluation electrodes that had a response ratio (the area under the curve) of ∼0.1 in a time window around the maximum of the second peak. These time windows had a width of 20–50 ms and the borders were set to such values to obtain this response ratio. In the example of Figure 2 the evaluation time window was set to 10–40 ms. We applied the following training protocol (slightly adapted from Shahaf and Marom [3]:

We stimulated the culture until the evaluation electrode showed at least 2 responses to the last 10 stimuli (response ratio ≥0.2) or until the maximum stimulation time of 10 min was reached. When the threshold (or the maximum stimulation time) was reached, stimulation stopped automatically (therefore, we use the term adaptive stimulation), followed by 5 minutes without stimulation. A sequence of such stimuli, followed by a 5 minutes period of no stimulation is called a cycle. We selected a stimulation electrode that induced network bursts upon most initial test stimuli. However, during some experiments the effectiveness of the stimulus decreased. Obviously, a deteriorating stimulus response will mask any possible learning effect. Therefore, we repeated the cycle until the network wide response to the stimuli dropped below threshold in three consecutive cycles. This threshold was set to 80% of the average response to the first 5 stimuli. A few early experiments were terminated when the threshold response ratio was reached immediately. These experiments lasted only several minutes and were not further described here. All following experiments were continued in such situations, until the network wide response dropped below threshold.

Using this criterion to finish the experiments, we performed 10 training experiments with a mean duration of just over 5 hours (316±160 min). We plotted the number of applied stimuli against the cycle number (a ‘learning curve’), and interpreted a decreasing number of stimuli as a learning effect. To evaluate the effects of the protocol on functional connectivity, we recorded at least one hour of spontaneous activity before and after the protocol. These spontaneous recordings were analyzed using conditional firing probabilities (section E)

### C. Training Experiments vs. Random Stimulation

We investigated if the training protocol had larger effects on connectivity than random stimulation at comparable frequencies, in periods of comparable duration. During training sessions, stimulation was switched on for 10 minutes or less, followed by 5 minutes without stimulation. Thus, during the training protocol, stimulation was switched on between ∼10% (desired response reached quickly) and 67% (desired response not reached) of the duration. To compare the effects of training sessions to those of random stimulation, we applied a 5 hour stimulation period between two spontaneous recordings of one hour each. During 40% of these 5 hours, slow electrical stimulation at 0.2 Hz was switched on. We stimulated at single electrodes which were randomly chosen before each next stimulus pulse. We estimated functional connectivity changes from the spontaneous recordings before and after manipulation as under B, and compared this to the changes after training sessions. In 7 experiments, we repeated this protocol with stimulation switched off to obtain an estimate for spontaneously occurring fluctuations..

### D. Fixed Electrode vs. Random Electrode Stimulation

Next, we investigated if the functional connectivity changes induced by single electrode stimulation depended on the stimulation electrodes. Therefore we first recorded spontaneous activity, then we stimulated for ∼1 hour, either permanently at one single electrode, or at an electrode which was chosen randomly before each stimulus pulse. Then we recorded spontaneous activity again. Both spontaneous recordings were used to assess induced changes in functional connectivity. For comparison with spontaneous fluctuation, we performed the same experiment with stimulation switched off.

### E. Connectivity Analysis

We used periods of spontaneous activity to analyze network functional connectivity. For all possible pairs of electrodes (60×59) we calculated conditional firing probabilities (CFPs) as the probability to record an action potential at electrode *j* at t = τ, given that one was recorded at electrode *i* at t = 0. If a CFP curve was not flat, the two neurons were functionally connected. An example is shown in Figure 2. This functional connection may be described by two parameters: strength (*M_i, j_*)and latency (*T_i, j_*) [15], which are obtained by fitting Equation 1. These parameters may be used to follow the development of a functional connection in time [18].
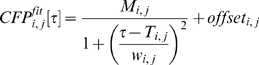
(1)To investigate the connectivity changes that resulted from either stimulation protocol (fixed electrode, random electrode or adaptive single electrode), we identified the set of persisting functional connections. That is the set of functional connections that were present in all data blocks before and after the stimulation protocol. All periods of spontaneous activity were divided into four or five data blocks. Thus, we had at least 4 values for strength and latency of all persisting functional connections before and after the manipulations to enable statistical comparison.

This analysis yielded two plasticity parameters: the number of significantly affected functional connections (as a fraction of the total number of persisting connections), and the relative change in magnitude of strength. To reduce these two to a single dimensional parameter, we calculated the plasticity index (PI) as the product of the fraction of significantly changed connections and the average magnitude of change. PI = 1 means that the strengths of all functional connections changed by 100%, PI = 0 means no changes at all.

We used Student's t-test to assess statistical significance of the differences found.

## Results

We applied the training protocol in 16 experiments. Selection of an electrode that induced network bursts upon most (>∼70%) stimulus pulses was never a problem. However, it appeared far more difficult to find a proper electrode for evaluation. If we used an evaluation electrode with an initial response ratio of 0.2 or higher, we were usually unable to obtain results that compared to those by Shahaf and Marom. In three cultures we did not find a suitable electrode for evaluation. Three other experiments were terminated prematurely because the network wide response to the driving stimulus decreased very quickly.

**Figure 4:**
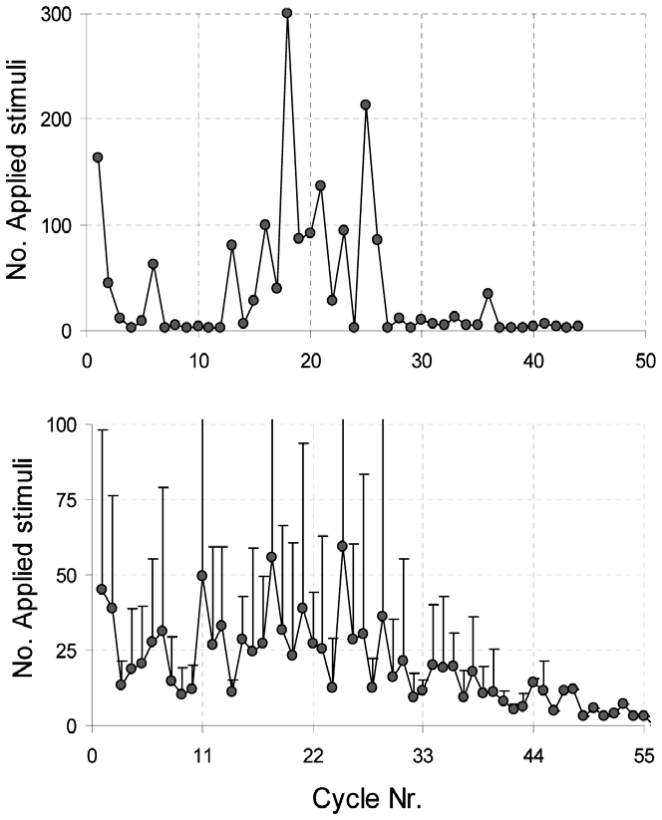
Development of the number of required stimuli during training experiments. **A** shows a typical example of an individual ‘learning curve’, as observed in 5 of 10 experiments. **B** depicts the average development (•, mean ± SD) of all 10 experiments. On average the number of applied stimuli decreases significantly with trial Nr. (Kendall's tau: Correlation coefficient: −0.33; P<0.01).

In 10 experiments we were able to find suitable electrodes for stimulation and evaluation. Figure 3 shows an example of the responses to the first 10 stimuli (gray dashed line) and to the last 10 stimuli (black solid line) of the training protocol in a successful experiment. In five experiments (50%) we obtained results as shown in the example of Figure 4A, which may be characterized by an initial decline (that continued for 14±8 cycles, mean ± SD), followed by a rise, roughly centered around trial Nr 20 (20±6). Eventually these learning curves reached a stable low level as in Figure 4A. The width of the increase around trial Nr. 20 varied and averaged 13±7 cycles.

In another 30% a stable low level was reached immediately, whereas 20% showed wild fluctuations without a clear trend. The average ‘learning curve’ of all cultures, including the 20% without a clear trend, is shown in Fig. 4B. The first 10 cycles yielded results very similar to the original results published by Shahaf and Marom [3]. Their paper presented only results of the first 10–12 cycles.

However, after the 10^th^ cycle the average number of stimuli often increased again. This phenomenon was seen in 5 individual experiments. Because the centre of this re-increase differed between experiments, on average the effect was somewhat blurred out, resulting in a lower and wider second peak in the averaged curve, as well as higher standard deviations (Fig 4B).

**Figure 5:**
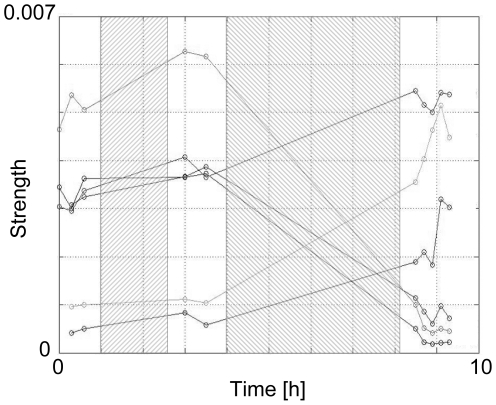
Strengths of persisting connections during one of our experiments. In this experiment there were only 6 persisting functional connections and the development of their strengths is represented by the 6 lines. The experiments had 5 phases. White areas: spontaneous activity recordings. left hatched area: random stimulation (see section II.B). Right hatched area: training protocol (see section II.C). The graphs illustrate that the strength of most individual connections was affected by the protocol. In total, the strength of 64% of all persisting connections was significantly changed. The figure also suggests that global parameters like mean strength may not be affected by the protocol.

Finally, we used spontaneous activity recorded before and after the training protocol to investigate functional connectivity changes in the network. Occasionally new functional connections appeared or existing ones disappeared during the training protocol, but on average this number was small, compared to the number of persisting functional connections. The strength of 65±24% of all persisting connections was significantly affected by the protocol (t-test, p<0.05), either up or down. Figure 5 shows an example with only very few persisting functional connections. Usually there were many more persisting connections (on average 360±209) and the depicted example is not representative in this respect. However, the selected example clearly shows that the strength of functional connections changed substantially, both up and down. Although the average strength of all persisting connections did not change, the mean *absolute* change was 55±33% (see Table 1), clearly exceeding the spontaneously occurring changes during a comparable time span (28±10%, in 34±14% of all persisting connections).

The mean absolute change did not differ from that after random stimulation. However, more functional connections were significantly affected 65±24% after training sessions, resulting in a plasticity index (PI, see Methods) that clearly exceeded the PI of a similar period of random stimulation (0.43±0.29 vs. 0.20±0.10).

Table 1 shows that the PI after the training protocol was significantly larger than after a similar period of random stimulation or no stimulation (t-test, p<0.05). The changes induced by random stimulation tended to be larger than spontaneously occurring ones. However, this difference was not significant (p = 0.08).

Next, we investigated the effect of slow stimulation on network functional connectivity in more detail. In 6 experiments we stimulated at one electrode using a low frequency (0.2–0.33 Hz), whereas in 6 other experiments, we used similar stimulation (periods and frequencies) at randomly changing electrode locations. Both types of stimulation yielded a similar fraction of significantly changed functional connections and the magnitude of changes agreed very well (Table 1). Both stimulations protocols yielded connectivity changes that were approximately twice as large as spontaneously occurring changes in a comparable period. The difference between random and no stimulation was significant (t-test, p<0.05), the connectivity changes due to single electrode stimulation were not significantly larger than spontaneous changes (p = 0.08).

Table 1 also shows that the mean absolute change (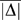) in both types of experiments was smaller than the mean absolute change in the training experiments. However, the periods of stimulation were also (much) shorter than during training sessions (∼1 hour vs. ∼5 hours).

We also calculated 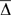, the average change in strength (see Table 1). In none of the experiments 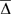 differed significantly from zero (t-test, all p>0.1), meaning that functional connections with increasing strength were always accompanied by others with decreasing strength.

## Discussion

In this study we aimed to repeat the experiments by Shahaf and Marom and investigated why these results appear so difficult to reproduce. We compared the effect of the proposed adaptive single electrode low frequency electrical stimulation to random stimulation at comparable low frequency and duration, and to spontaneously occurring functional connectivity changes in networks during such a period.

Effective electrical stimulation usually induces a network reaction that may be characterized by an early response (up to ∼20ms), reflecting mostly direct activation of a distinct subset of neurons, followed by a late response lasting up to hundreds of milliseconds [7], [19], [24]–[26]. This late ‘reverberating’ phase involves propagation of signals through multiple, and probably recurrent synaptic pathways [1]. If a culture is frequently stimulated, even at low stimulation rates, the network response often decreases. Jimbo et al showed that this response usually decreased when the stimulus frequency exceeded ⅓ Hz [27]. Based on this study we did not use stimulation frequencies higher than ⅓ Hz. Eytan et al. reported considerable fluctuations in the network response at frequencies above 0.1 Hz. Moreover, at ⅓ Hz, they found a negative trend in the response [1]. In some of our experiments we saw a decreasing response at this frequency. If this occurred, we lowered the frequency to 0.2 Hz.

The response to the stimulus is critical in the training experiments. Obviously, the impact of a single stimulus pulse is much larger if it triggers a network burst. Also, the probability to record an action potential at the evaluation electrode in the set time interval is much higher if a stimulus pulse triggers a network burst. We therefore used the network wide response as a measure to determine when to terminate the experiments.

The applied training protocol was effective in 50% of our experiments. It may be argued that the protocol was also effective in the 30% of our experiments that immediately reached a stable low level. This percentage could have been higher if some experiments were not terminated early if a response reached the threshold immediately. A network may produce the desired output in the first cycle by sheer chance, and thus experience no further drive to explore other connectivity. However, here the results were more difficult to interpret because the ‘learning curves’ did not show any improvement. In 20% the training protocol did not reduce the number of input stimuli needed to reach the desired output. Still, in all experiments, including these unsuccessful ones, analysis of spontaneous activity before and after the protocol showed that the strength of a major part of all functional connections had changed. This means that the training protocol always did affect functional network connectivity, even though it sometimes failed to induce a chosen modification.

One possible explanation for the highly varying success of the training protocol is the balance that cultured networks may develop between activity and connectivity. Because activity patterns arise from certain connectivity, and activity, in turn, influences connectivity, the finding that networks develop stable activity patterns [10], [15] may be interpreted as an established balance between activity and connectivity. If external stimulation pushes the network out of balance, it may develop towards a new equilibrium which may or may not include the selected connection.

Thus the choice of the functional connection to be trained may determine the success of the protocol, but one cannot predict whether or not a selected connection will lead to a success. On average, however, we did find a significantly ‘improved’ response to electrical stimuli after the training protocol.

Another interesting phenomenon is the rising ‘learning curve’ around cycle 20, after an initial decrease. Because it was observed in 50% of the experiments, it seems unlikely that this occurred purely coincidentally. This is emphasized even more by the fact that in a later paper Marom et al observed a similar increase around stimulation cycle 18 in an averaged learning curve of 16 experiments [19]. Unfortunately, the scale of the graph (Fig. 10 in their paper) was adapted to include another curve, which masked the effects, and it is not further addressed in their paper. This second peak in the learning curves might be caused by a reorganization of the whole network. We hypothesize that initially an individual connection can be changed, while the network is in an unbalanced state, and that internal forces will then drive the network into a new balance that may or may not contain the alteration of the selected functional connection. In the 30% group that immediately reached a stable low level, the new balance may have contained the chosen alteration of the selected connection, whereas in the 50% group with a second peak in their learning curves, there may have been a conflict between intended change and newly found balance. This would explain why it took so much longer before a stable low level was reached. It is even possible that the ‘learning curves’ of the 20% unsuccessful experiments would eventually have reached a stable low level if we could have continued measurements long enough. What we can conclude is that the learning protocol did affect functional network connectivity in all experiments; *on average* we found a descending learning curve. However, individual curves usually did not descend very smoothly, suggesting a more complicated mechanism than just strengthening of a synaptic pathway.

Figure 5 suggests that random stimulation (left hatched bar) hardly affected connectivity. However, on average, the random stimulation period lasted much shorter (usually 1–1½ hours) than the training protocol (∼5 hours). During training sessions, stimulation was switched on for 10 minutes or less, followed by 5 minutes without stimulation. To compare the effects of training sessions to those of random stimulation, we applied a 5 hour stimulation period between two spontaneous recordings. During 40% of these 5 hours, stimulation was switched on, see Methods. We found that the fraction of significantly changed functional connections was far larger after the training protocol (65±24%) than after random stimulation (35±10%), see Table 1. The magnitude of changes was equal to those after the training protocol. To summarize all plastic changes into a one dimensional parameter, we calculated the plasticity index (PI, see Methods). The training protocol yielded a significantly higher PI than similar periods of random stimulation (0.43±0.29 vs. 0.20±0.10).

Finally we compared functional connectivity changes after slow stimulation through a randomly varying electrode to those after slow stimulation at one selected electrode. Table 1 shows that both the number of significantly changed functional connections, as well as the average magnitude of changes, and thus also PI's, were very similar. Apparently, the site of stimulation does not affect the plasticity index. This does not mean that stimulation at another electrode induces the same functional connectivity changes, it is well possible that other connections will change when another electrode is stimulated, only the magnitude of changes are equal.

Our data show that slow stimuli do affect functional network connectivity in all applied protocols. It seems unlikely that the stimulus it self will have such an impact on connectivity, rather, the resulting network bursts may change connectivity.

Several studies used tetanus stimulation to induce connectivity changes, based on the well established tetani used to potentiate synapses in intra-cellular experiments, often in hippocampal neurons. This technique appeared to be useful in extracellular experiments on MEA's as well [2], [4], [28]. In a recent paper Chiappalone et al. found a fairly limited effect of tetanic stimulation on single pulse evoked spiking activity, which could be greatly increased when paired with a ‘weak’ stimulation (single pulses at a rate of 0.2 Hz) [7]. Moreover, given the difficulties that Wagenaar et al had, trying to demonstrate induced plasticity using tetani [5] one may question their effectiveness. Chiappalone et al also suggested that low frequency stimuli did not produce any changes in the stimulus response of networks. However, they first verified the stability of this response across 30–40 minutes periods of slow stimulation and cultures that failed this stability test (13% of their experiments) were not used for further analysis. Furthermore, they did not investigate individual connections, but looked at a more global parameter: network wide stimulus response, and they only considered changes >20% to be significant. Our results indicate that 1 hour of stimulation (1½–2 times longer than their stimulation periods) yielded a plasticity index around 0.11 (see Table 1). This was twice as high as that after one hour without stimulation (PI = 0.05), but may well have gone undetected in their more global analysis.

Vajda et al used low frequency pulses to stimulate a culture (1 hour, 40% of that period stimulation was switched on) and were able to induce changes in initially stable patterns of stereotypical spontaneous activity, as observed in changes in single site and culture wide network activity as well as the spatio temporal dynamics of network bursting [6]. Although they used single electrode stimulation at 6 consecutive electrodes (in between our single or random electrode stimulation), their results can be compared to ours, because we did not find significant differences between these two stimulation protocols. They suggested that connectivity changes induced by slow electrical stimulation could be caused by: 1: plasticity, 2: changed intrinsic neuronal properties such as excitability, or 3: transition from one attractor state to another. If one assumes that a slight change in one of the pathways may already lead to a newly developed balance, the first and the third hypothesis become very similar. Our work supports this view. Our hypothesis is that: 1) low frequency stimulation is not necessarily less intense than tetanic stimulation, as long as it frequently induces network bursts 2) Networks seem to develop an activity ↔ connectivity balance, and stimulation may push the network out of the equilibrium. Networks will then develop towards a new balanced connectivity. Thus, slow electrical stimuli may trigger internal network forces to induce connectivity changes and are therefore powerful stimuli. Stimulation leads to network bursts, which originate at other points than a spontaneously occurring burst might have, and spreading of activity may follow alternative pathways. Although a recent study by Eytan and Marom suggested that a different stimulation site did not change the role of ‘privileged neurons’, a certain set of neurons that usually fire shortly before or during the onset of a network burst, their results do indicate that stimulation triggers a larger set of neurons to fire in the early phase of a burst, thereby activating pathways that might be left unused without stimulation [29]. Therefore electrical stimulation may lead to different timing in firing patterns of neurons, thus disturbing the activity ↔ connectivity balance. Our results show that it does not really matter where a culture is stimulated; randomly varying stimulation sites induce comparable connectivity changes as a single selected stimulation electrode. Apparently, pushing the network out of balance leads to connectivity changes. Then, the network will find a new equilibrium. The applied stimuli do not determine this new balance, they are only the trigger to develop a new equilibrium. Yet we can not determine whether different stimuli lead to different new connectivities. This would be an important finding, as it might explain how the brain deals with parallel memories. Each time something is learned, it develops a new connectivity, which incorporates the newly learned facts, and combines it to what was already stored.

In summary, the training protocol as proposed by Shahaf and Marom yielded functional connectivity changes that were significantly larger than those obtained after random stimulation during a period of comparable duration or spontaneous plasticity during such periods. We found no difference between functional connectivity changes due to single or random electrode stimulation. Connectivity changes after either stimulation protocol were larger than after an equal period without stimulation. We may therefore conclude that slow electrical stimulation at a single electrode did affect functional network connectivity. The changes induced by the training protocol significantly exceeded those induced by the other stimulation protocols.

The extra change must be caused by the adaptive character of the stimulation. Shahaf and Marom demonstrated that their protocol without feedback did not result in a declining ‘learning curve’. Thus, adapting the stimulus to the network response enables larger connectivity changes, as well as a declining learning curve. We hypothesize that adaptive stimulation may force networks to a new balance, at a larger distance from the initial state, because it no longer accepts any arbitrary new equilibrium, but continues to drive exploration until a balance is found within a certain restricted subspace.
